# Mono(ADP-ribosyl)ation Enzymes and NAD^+^ Metabolism: A Focus on Diseases and Therapeutic Perspectives

**DOI:** 10.3390/cells10010128

**Published:** 2021-01-11

**Authors:** Palmiro Poltronieri, Angela Celetti, Luca Palazzo

**Affiliations:** 1 Institute of Sciences of Food Productions, National Research Council of Italy, via Monteroni 7, 73100 Lecce, Italy; 2 Institute for the Experimental Endocrinology and Oncology, National Research Council of Italy, Via Sergio Pansini 5, 80131 Naples, Italy; 3 Institute for the Experimental Endocrinology and Oncology, National Research Council of Italy, Via Tommaso de Amicis 95, 80145 Naples, Italy

**Keywords:** ADP-ribosyl transferase (ADPRT), Mono ADP-ribose transferases (mARTs), cholera-toxin-like ARTs (ARTCs), Diphtheria-toxin-like ARTs (ARTDs), Sirtuins (Sirt), NAD precursors

## Abstract

Mono(ADP-ribose) transferases and mono(ADP-ribosyl)ating sirtuins use NAD^+^ to perform the mono(ADP-ribosyl)ation, a simple form of post-translational modification of proteins and, in some cases, of nucleic acids. The availability of NAD^+^ is a limiting step and an essential requisite for NAD^+^ consuming enzymes. The synthesis and degradation of NAD^+^, as well as the transport of its key intermediates among cell compartments, play a vital role in the maintenance of optimal NAD^+^ levels, which are essential for the regulation of NAD^+^-utilizing enzymes. In this review, we provide an overview of the current knowledge of NAD^+^ metabolism, highlighting the functional liaison with mono(ADP-ribosyl)ating enzymes, such as the well-known ARTD10 (also named PARP10), SIRT6, and SIRT7. To this aim, we discuss the link of these enzymes with NAD^+^ metabolism and chronic diseases, such as cancer, degenerative disorders and aging.

## 1. Introduction

Nicotinamide adenine dinucleotide (NAD^+^) is an essential pyridine nucleotide cofactor that is crucial for the activity of numerous enzymes involved in fundamental cellular processes, such as cellular energy metabolism and adaptive response to stress conditions. Being NAD^+^, a limiting factor for the activity of dehydrogenase and NAD^+^-utilizing enzymes, such as (ADP-ribosyl) transferases (ART), Sirtuins (Sirt) and NAD^+^-dependent histone deacetylases, the availability of NAD^+^ and an optimal NAD+/NADH ratio govern vital cellular redox and enzymatic reactions, including mitochondrial biology, energy production, metabolism, DNA repair, epigenetic modulation of gene expression, apoptosis and intracellular signaling [1]. Thereby, NAD^+^ metabolism has a key role in the maintenance of cellular physiology and predisposition to a wide range of chronic diseases [2].

ARTs, which include the superfamily of poly(ADP-ribose) polymerase (PARP) enzymes, contribute significantly to the reduction of cellular NAD^+^ supply. Indeed, NAD^+^ depletion easily occurs in response to excessive DNA damage, which is one of the well-known activation mechanisms of the main PARPs involved in DNA damage repair, namely PARP1 and PARP2 (also named ARTD1 and ARTD2, respectively) [3,4,5]. Specifically, ARTs utilize NAD^+^ as a donor to transfer single or multiple units of ADP-ribose to substrate molecules, which can be proteins and, in some cases, nucleic acids [6,7,8,9,10,11]. Such enzymatic reaction is reversible and takes the name of ADP-ribosylation, a type of post-translational modification (PTM) [12,13]. Two forms of ADP-ribosylation are described, the poly(ADP-ribosyl)ation (abbreviated as PARylation), determined by polymers of ADP-ribose covalently linked onto target substrates, and mono(ADP-ribosyl)ation (abbreviated as MARylation), consisting in a single ADP-ribose unit modification [14,15]. Historically, three classes of enzymes form the ART superfamily; the diphtheria-toxin like transferases (ARTD), which includes PARPs, the cholera-toxin-like transferases (ARTC), and a subgroup of Sirt, a NAD^+^-dependent histone/protein deacetylases belonging to HDAC group III, which are considerate the writers of ADP-ribosylation reactions [16,17,18,19,20,21].

The ARTDs/PARPs branch of ARTs can be further grouped in two categories based on their ability to perform MARylation and PARylation (the latter will not be discussed in this review) [14,20,22,23,24,25]. Herein, we will discuss mammalian enzymes responsible for protein MARylation, and their pathophysiological functions by focusing on the potential of inhibiting their enzymatic activities in order to provide novel therapeutic perspectives in chronic human diseases. In this regard, the impact of NAD^+^ homeostasis and the potential of supplementing NAD^+^ precursors to exogenously regulate ADP-ribosylation reactions will be additionally discussed.

## 2. NAD^+^ Biosynthetic and Salvage Pathway: Links between NAD^+^ Levels and Diseases

### 2.1. Enzymes of the NAD^+^ Synthesis and NAD^+^ Salvage Pathway

**Figure 1:**
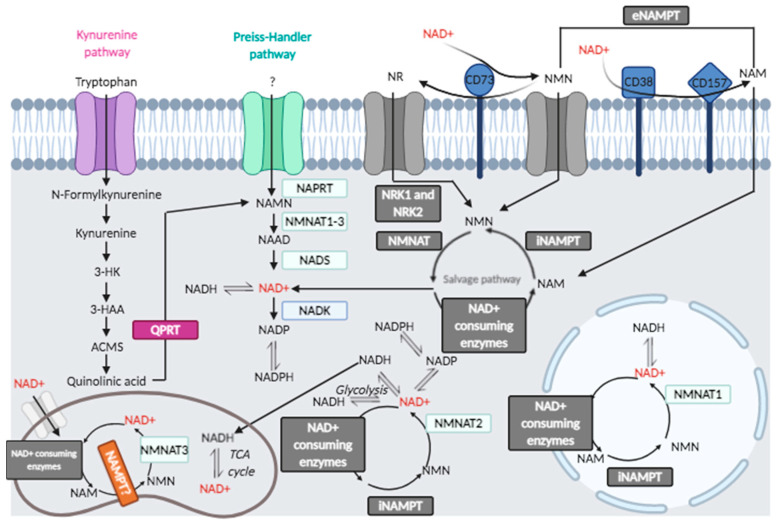
Cellular and extracellular NAD^+^ metabolic pathways.

Localized compartmentalization of NAD^+^-synthesizing and NAD^+^-consuming enzymes allows for the steady state of NAD^+^ levels in cells [26,27]. NAD^+^-metabolic pathways are summarized in Figure 1. Although connected with another, different subcellular pools of NAD^+^ are independently regulated, and free NAD^+^ levels tightly control signaling by ARTs as well as redox metabolism (see Section 3) [27]. Importantly, a growing number of reports support NAD^+^ metabolism as a major therapeutic target for age-related diseases.

#### 2.1.1. Kynurenine Pathway

De novo synthesis of NAD^+^ in cells, called the kynurenine pathway, originates from tryptophan and involves quinolinate phosphoribosyltransferase (QPRT). Interestingly, NAD^+^ precursor kynurenine has been used in some trials to study the replenishment of NAD^+^ pools under stress conditions [26].

#### 2.1.2. NAD^+^ Salvage Pathway, Membrane-Associated and Extracellular Sources of NAD^+^

Nicotinamide (NAM), the product of enzymes that use NAD^+^ as cofactor/substrate, is the starting substrate in the NAD^+^ salvage pathway [28], predominant in maintaining NAD^+^ levels in many cells. One pathway involves nicotinic acid phosphoribosyltransferase domain-containing 1 (NAPRT1) starting from nicotinic acid (NA) (niacin, a form of vitamin B3). NA is supplied by diet and metabolized by NAPRT1 to nicotinic acid mononucleotide (NAMN) that is transformed into NAD^+^ in the Preiss–Handler pathway. A second pathway involves nicotinamide phosphoribosyltransferase (NAMPT), which produces nicotinamide mononucleotide (NMN) from nicotinamide (NAM) to which 5-phospho-α-D-ribosyl 1-pyrophosphate (PRPP) donates the ribose group, a reaction that consumes ATP [29]. In neurons, NAMPT orchestrates the NAD^+^ salvage pathway and resides in the mitochondrial matrix [30]. NAMPT expression decreases during treatment of APP/PS1 mice with its inhibitor, FK866, and is rescued by supplementation with NAD^+^, as reported [26].

Nicotinamide mononucleotide adenylyltransferases (NMNATs), also known as NAD^+^ synthases, convert NMN to NAD^+^. The supplementation with NAD^+^ precursors (NMN, NR) or overexpression of NMNAT1 shows beneficial effects in neurodegenerative diseases. NAD^+^ is produced in each compartment by different NMNAT isoforms [31]. In eukaryotic cells, there are three isoforms of NMNAT (namely, NMNAT1, 2 and 3). NMNAT1 is localized in nuclei, NMNAT2 in cytosol, and NMNAT3 is mitochondrial. NMNAT3 spliced form, FKSG76, is not essential to maintain NAD^+^ levels in mitochondria and cleaves NAD^+^ more than acting on NMN as substrate; thus, the reverse reaction is more favorable than NAD^+^ synthesis [31]. NMN production with NAD^+^ degradation has also been reported for NMNAT2 [31]. Thus, other NMNATs may have a major role, such as NMNAT1, indicating the main involvement of the nuclear compartment and the resident NMNAT1 in restoring NAD^+^ levels.

The extracellular NAD^+^ sustains the mitochondrial NAD^+^ pool in an ATP-independent manner: eNAD^+^ is degraded by the extracellular cluster of differentiation 38 (CD38) and CD73 to form the metabolic precursors NMN, nicotinamide riboside (NR), NAM and nicotinic acid (NA). Nicotinamide nucleotide transhydrogenase (NNT) sustains mitochondrial NAD^+^ pool through the shuttling of reducing groups from the cytosolic NADH, as discussed in the next paragraphs.

NAD^+^ boosting molecules are recognized as effectively improving age-related diseases [32,33]. Nicotinamide riboside (NR) is an additional salvage pathway element and NAD^+^ precursor. NR is converted to NMN by nicotinamide ribose kinases, NRK1 and NRK2. Nma1/Nma2 converts NMN to NAD+, which utilize either NMN or nicotinic acid mononucleotide (NaMN) [34]. NR can give rise to NAM by purine nucleoside phosphorylase (NP), and after NAM modification to NMN, NMNAT converts NMN to NAD^+^ [31]. NR can enter into cells by Nrt1 transporter; thus, NR can be supplemented with diet or drugs. NR does not produce side effects or flushing [34]. Furthermore, NR-reduced form, NRH, can be supplemented as NAD^+^ precursor, being NRH more stable than NR in plasma, and can be converted to NAD^+^ through the NMNH intermediate, with adenosine kinase (ADK) acting as an NRH kinase [35]. Vacor adenine dinucleotide (VAD), a mimetic of NAD^+^, causes inhibition of NMNAT2 and NMNAT3, but not of NMNAT1 [36,37]. Previous results have shown the coordinated requirement of NAMPT and NMNAT2 in NAD^+^ synthesis and how their inhibition could lead to derangement of the NAD^+^ pool. There are structural and functional similarities between human NAMPT and nicotinate phosphoribosyltransferase (NAPRT), which uses nicotinic acid (NA) to produce nicotinic acid mononucleotide (NaMN) [38]. Using FK866 to inhibit NAMPT, A375 cells were treated with nicotinamide, nicotinic acid, nicotinamide riboside, kynurenine and quinolinic acid as precursors of NAD^+^, showing that each substrate has organelle-specific ability to rescue from NAMPT block [38]. The authors showed that cytosolic NAD^+^ content decreased, mitochondrial NAD^+^ did not change, so that NAMPT was not affected in the mitochondrial compartment [38], whereas nicotinamide riboside kinase (NRK) was found active in nuclei and in mitochondria, and NAPRT was predominant in cytosol and mitochondria [39]. NAMPT and NAPRT can be secreted extracellularly (eNAMPT, eNAPRT) as cytokines and damage-associated molecular patterns (DAMPs): eNAMPT is known as pre-B cell colony-enhancing factor or visfatin [40], acting through the activation of Toll-like receptor 4 (TLR4) [41]. The enzyme is involved in human inflammation, obesity, diabetes and has been indicated as a target for anticancer and immunotherapy strategy [41]: moreover, eNAPRT is a biomarker of sepsis and septic shock [42].

Ectonucleotidases form two groups based on substrate specificity: one group includes enzymes that metabolize extracellular NAD^+^, such as CD38, CD157, and CD203 (also known as ENPP1); the latter can also hydrolyze protein ADP-ribosylation [43,44]; the second group consists of enzymes that degrade ATP, such as the ecto-nucleoside triphosphate diphosphohydrolase CD39 (E-NTPDase) and the ecto-5′-nucleotidase/cluster of differentiation 73 (CD73) [43,44,45,46].

Adenine nucleotides (AdNs) have an important role in immunity and inflammation [47,48]. Intracellular AdNs originating from ATP or NAD^+^ are signaling molecules in immune cells, such as T lymphocytes, macrophages, microglia and astrocytes. The ectoenzyme CD73, expressed on multiple cells [47], catalyzes the conversion of purine 5′-mononucleotides to nucleosides, such as adenosine and dephosphorylates NMN, so that the reaction product, NR, can be taken up into the cells [48].

An NAD^+^ utilization pathway relies on 1-methylnicotinamide (MNAM), synthesized by nicotinamide-N-methyltransferase (NMMT), a metabolite with pleiotropic effects [49], including Sirt activation, lifespan extension, induction of nitric oxide, decrease in prostacyclins. Furthermore, MNAM is oxidized by aldehyde oxidase (AOX) to produce H_2_O_2_, regulating ROS-dependent responses [50].

NAD^+^-consuming enzymes require constant NAD^+^ production, which is supported in mitochondria by the membrane protein nicotinamide nucleotide transhydrogenase (NNT). NNT maintains a high NAD^+^/NADH ratio, has a role in determining the metabolic state and constitutes a potential tool to mimic calorie restriction and to slow aging [51]. A putative therapeutic role has been assigned to NNT in counteracting mitochondrial dysfunction arising from ROS damage. Mitochondria generate ATP via the Krebs cycle, oxidation of fatty acids, and oxidative phosphorylation (oxphos). These activities make mitochondria the principal source of reactive oxygen species (ROS) within the cell. The NADPH pool is maintained by the combined action of NNT, malic enzyme, and isocitrate dehydrogenase 2 (IDH2). The IDH2-catalyzed reaction is driven by the NADPH-reduction potential, which in turn is provided by the proton motive force-dependent "forward" NNT reaction. NNT can change its "forward" direction to a "reverse" catalysis under pathological conditions to maintain the NAD(P)^+^/NAD(P)H ratio in the mitochondrial matrix. The reverse Krebs cycle is essential in proliferating cells [51]. Mitochondria perform oxidative decarboxylation of isocitrate to α-ketoglutarate (α-KG). During glutamine oxidation, the reductive carboxylation of α-KG in the reverse direction of the Krebs cycle is an essential metabolic pathway for pluripotent stem cell survival. SIRT4–mediated modification of glutamate dehydrogenase blocks α-KG production: the temporary modification may decrease glutamine metabolism.

### 2.2. NAD^+^ Availability in Aging and Disease

The NAD^+^-dependent activity of mARTs and MARylating Sirt regulates energy metabolism and maintains cellular homeostasis in response to physiological stress responses, thus preventing several age-related diseases. Therefore, stimulation of NAD^+^-dependent enzyme activities by providing the precursors for NAD^+^ synthesis of small molecule activators of NAD^+^ salvage pathway enzymes would be beneficial to human health, positively connecting with the NNT forward mode that ensures a high NAD^+^/NADH ratio [51]. An increase in NAD^+^ availability was shown to extend lifespan and to improve several disease states [52].

Mitochondrial dysfunction, imbalance of NAD^+^ and ATP local pools, and oxidative stress are prominent features of various typologies of human diseases, including neurodegenerative disorders and cancer. Cellular NAD^+^ concentrations decrease during aging, as well as the expression of enzymes of the NAD^+^ salvage pathway. NAMPT expression levels decline with aging [53]: NAMPT depletion aggravates, while NAMPT overexpression prevents age-related changes [53]. NAMPT activation requires the deacetylase activity of SIRT6 and modulation by AMPK; thus, a block in any of these modifications may be relevant in NAD^+^ salvage. CD38 is reported to increase in aged individuals [53], with consequences on increased NAD^+^ consumption, which is linked to oxidative stress [27]. Brain hypoperfusion appears to induce oxidative stress (OS), largely due to reactive oxygen species (ROS), and, over time, driving mitochondrial failure, an initiating factor of Alzheimer’s disease (AD) [54]. Mitochondria play a critical role in viability and death in neurons and neuroglia since they regulate energy and oxygen metabolism as well as cell death pathways [55]. Thus, defects in NAD^+^ metabolism has been proposed as a hallmark of metabolic diseases (obesity, diabetes, dyslipidemia, non-alcoholic fatty liver disease) as well as for neurodegeneration [55,56,57]. It has been proposed that the modulation of NAD^+^ levels is an important element to control metabolism, either in health or in disease [18]. There is an axis linking NAD^+^ with Sirt in aging and disease [17], as well as linking NAD^+^ and ARTDs/PARPs, showing the beneficial effects of using PARP inhibitors (relevant ART inhibitors are discussed in Section 3.1.2 and listed in Table 1) [58,59,60,61,62].

SARS-CoV2 (COVID-19) infection was shown to affect NAD^+^ metabolome, altering the expression of several ARTDs and of NAMPT enzyme [78]. Indeed, the increased NAD^+^ levels may improve the immune response to the virus, as reported [79]. It may be important to investigate the link between mART enzyme activity and NAD^+^ levels and test mART inhibitors in disease models. 

## 3. Mono(ADP-Ribosyl) Transferases (mART)

### 3.1. Diphtheria-Toxin-Like Mono(ADP-Ribosyl) Transferases

The majority of human ARTs belonging to the ARTD/PARP superfamily possess MARylation activity and include: ARTD3/PARP3 [80,81,82,83], ARTD4/PARP4 [84], ARTD7/PARP15 [84], ARTD8/PARP14 [85,86], ARTD10/PARP10 [87], ARTD11/PARP11 [88], ARTD12/PARP12 [89], ARTD14/PARP7 [90], ARTD15/PARP16 [91], ARTD16/PARP8 [92,93], and ARTD17/PARP6 [94]. By contrast, only four ARTD/PARP enzymes perform PARylation, namely: ARTD1/PARP1, ARTD2/PARP2, ARTD5/PARP5a (frequently called Tankyrase-1), and ARTD6/PARP5b (known as Tankyrase-2).

In the past years, the major contribution to the understanding of ADP-ribosylation reactions has been provided by studies on ARTD1/PARP1- and ARTD2/PARP2-dependent PARylation performed under stress conditions [3,4], considered to be the major source of NAD^+^ consumption. By contrast, the investigation of MARylation reactions has suffered major technical limitations, such as the lack of antibodies to visualize this modification. It is important to determine whether the activity of ARTs that catalyze MARylation are regulated by changes in the levels of free NAD^+^ in the subcellular compartment in which they are restricted; equally important is to understand how NAD^+^ consumption activities by ART localized in different cellular compartments may influence the activity of other NAD^+^ consumers and redox enzymes in a different compartment. In order to address these issues, an in-depth understanding of ART biochemical and cellular features, as well as of proteins modified by ADP-ribosylation, is needed.

Studies focused on the investigation of ARTD1/PARP1 and ARTD2/PARP2 mechanisms of DNA damage repair uncover serine residues of target proteins as the most abundant acceptor sites for PARylation [11,95,96,97,98,99,100,101], with a minor contribution of tyrosine, lysine, and acidic residues [101,102]. By contrast, for MARylation, which is the major ADP-ribosylation under physiological conditions, the most abundant target residues are depicted by arginine, cysteine, and histidine [102,103,104]. Nevertheless, the real contribution of enzymatic ADP-ribosylation vs. nonenzymatic conjugation reactions is in need of further investigation [105,106,107].

A large number of target proteins are known as acceptors of ARTD enzymes with MARylating activity, which under certain circumstances could become dominant NAD^+^ consumers.

ARTD3/PARP3 has been involved in cellular response to DNA damage and mitotic progression, for instance, through the modification of the mitotic spindle components NuMa1 and ARTD5/PARP5a, the DNA repair proteins Ku80 and ARTD1/PARP1, and the histone H2B [82,108,109]. ARTD8 MARylates HDAC2 and HDAC3, the histone deacetylases involved in the epigenetic regulation of chromatin, TBK-1, an inhibitor of interferon synthesis, and STAT1 [110], consequently reducing STAT1 phosphorylation and suppressing the IFNγ–STAT1 signaling and the TNF-α/IL1-β proinflammatory pathway in macrophages. ARTD8 enhances histone acetylation to promote transcription of IFN-I genes [110].

N-(2(-9H-carbazol-1-yl)phenyl)acetamide (GeA-69) was identified as a novel allosteric ARTD8 inhibitor [111]. Recently, a role for the ADP-ribosyltransferase 8 (ARTD8) has been identified in the dynamics of the DNA replication controlled by ATR [112], as it modulates the response to ATR-CHK1 pathway inhibitors. ARTD8 interacts with PCNA, a DNA replication machinery component, promoting replication of DNA lesions and common fragile sites [113]. By using an engineered ARTD8/PARP14 variant, Carter-O’Connell and colleagues [114] identified 114 specific MARylation targets, several of which are RNA regulatory proteins. Interestingly, one of these targets is a catalytic inactive ARTD, namely ARTD13/PARP13, which is known to play a role in regulating RNA stability [8,84,115,116,117]. Then, ARTD8 MARylates ARTD13 on several acidic amino acids [114], whose biological outcome still must be addressed. Indeed, mutation of ARTD13 amino acids modified by ARTD8 does not affect RNA-binding functions. Interestingly, ARTD8 automodification sites have been mapped on tyrosine and histidine sites [102], thus raising questions about the real amino acid specificity of this enzyme. ARTD17/PARP6, considered as a tumor suppressor, limits the proliferation and spreading ability of the hepatocellular carcinoma cells by degrading XRCC6/Ku70 and by regulating the Wnt/ß-catenin pathway [118]. The ubiquitin ligase HDM2 can interact with ARTD17 and XRCC6. The recent identification of ARTD17 as a regulator of dendrite morphogenesis supports a role for MARylation in neuron development [94]. Expression of wild-type ARTD17 increased dendritic complexity; conversely, the expression of a catalytically inactive ARTD17 mutant or a cysteine-rich domain deletion mutant with a reduced catalytic activity decreased dendritic complexity [94].

ARTD11 targets NXF1 (an mRNA-binding protein involved in the nucleocytoplasmic shuttle) and NUP98, while modification of NAGK is dependent on the WWE domain present in the ARTD11/PARP11 enzyme [119,120]. ITK7, a quinazolin-4(3H)-one scaffold with propynyl in R1 and pyrimidine in R2 substitutions, showed high specificity toward ARTD11, whose activity towards nuclear pore complex proteins leads ARTD11 to dissociate from the nuclear envelope [121]. ARTD11 was also reported to target ubiquitin E3 ligase β-transducin repeat-containing protein (β-TrCP), promoting IFNα/β receptor subunit 1 (IFNAR1) degradation [88].

Membrane-anchored ARTD15/PARP16 MARylates karyopherin-1-β (Kapβ1), which interacts with importin-α/karyopherin-α (Kapα) [122]. Kapβ1 molecules can also transport cargoes independently of Kapα. Both exportin1, also known as Chromosomal maintenance 1 (Crm1), and Kapß1 have the potential to be regarded as biomarkers and therapeutic targets, as the inhibition of Kapß1 expression in cervical cancer cells leads to apoptotic cell death, suggesting a functional dependency on Kapß1 overexpression for cervical cancer cells transformation ability. Thus, the upregulation of either the importer or exporter components of nucleo-cytoplasmic trafficking may result in efficient transport, which can sustain the high proliferation rate of the cancer cells. Whereas normal epithelial and fibroblast cells are unaffected by Kapß1 inhibition, cancer cells die as a consequence of Kapß1 inhibition. Moreover, Kapß1 has a role in ER stress and unfolded protein response (UPR). ARTD15 MARylates PKR-like endoplasmic reticulum kinase (PERK) and inositol-requiring enzyme 1 α (IRE1α), two key stress sensors in the UPR in the endoplasmic reticulum. These findings link ARTD15 to inflammation showing that the UPR-linked inflammation is involved in the pathogenesis of inflammatory diseases [91]. Through Kapß1 MARylation, ARTD15 may represent a novel, crucial element in the regulatory mechanism of nucleo-cytoplasmic trafficking. In this respect, both the site of ADP ribosylation on Kapß1 and the ARTD15 catalytic site can be envisaged as potential targets for innovative therapeutic strategies.

ARTD9/PARP9 recently joined the family of MARylating enzymes. Indeed, it has been listed as an inactive ARTD until recently. Yang and colleague [123] reported ARTD9 to heterodimerize with DTX3L, a histone E3 ligase involved in DNA damage repair catalyzing the NAD^+^-dependent MARylation of ubiquitin molecules, at the carboxyl group of ubiquitin Gly76. As Gly76 is normally used for ubiquitin conjugation to substrates, ADP-ribosylation of the ubiquitin precludes ubiquitylation reactions [123]. The DTX3L/ARTD9 complex has also been found involved in the regulation of numerous processes, such as the ubiquitination of histones (such as histone H2BJ) and viral proteases (specifically viral 3C proteases), and the interferon-driven ubiquitination signaling able to control viral infections [124]. It also has been regarded as a component of a ubiquitinating LPS-responsive protein complex suggesting a role in LPS-mediated macrophage activation [125]. Surprisingly, Chatrin and colleagues [126] showed that the ART activity on ubiquitin’s Gly76 is not provided by ARTD9 but by the conserved carboxyl-terminal RING and DTC (deltex carboxyl-terminal) domains of DTX3L and other human deltex proteins (DTX1 to DTX4). Indeed, Yang and colleagues [123] made their observation in the context of DTX3L/ARTD9 heterodimer, but not of DTX3L on its own. Thus, this milestone finding first suggests that ARTD9 putative enzymatic activity remains elusive and further add Deltex proteins as novel NAD^+^-dependent transferases.

In addition to protein substrates of mART, several observations suggest that nucleic acids, both DNA and RNAs, can be ADP-ribosylated. In this regard, ARTD10/PARP10, as well as ARTD11/PARP11, ARTD7/PARP15, and the divergent PARP homolog TRPT1, also named PARP18, can ADP-ribosylate phosphorylated ends of RNA [8,127]. Originally, MAR/PAR modification of nucleic acids was studied for DNA. ARTD1, ARTD2 and ARTD3 modify the 5’-phosphate group of DNA ends to repair damaged DNA ends [83,128]. The phosphate ends of DNA duplexes or the single-stranded oligonucleotides can be PARylated. Eukaryotic RNA 5’-end sustains various forms of capping. NAD^+^ capping and de-NADding is one of the modifications related to NAD^+^ homeostasis [129]. NAD^+^ consumption in nuclear compartments may affect NAD^+^ availability for all the enzymes dependent on NAD^+^ for modification of proteins and of nucleic acids, including both MARylation of proteins and RNAs and RNA NADdylation. Nicotinamide (NAM) is recycled in the NAD^+^ salvage pathway in cells under physiological conditions, while during aging and in chronic conditions, most of the NAD^+^ salvage pathway enzymes are decreased or downregulated. Reduced NAD^+^ availability under these conditions may affect both the NAD^+^ dependent enzymes operating in mitochondria as well as those residing in the other cell compartments.

#### 3.1.1. ARTD10 Functions

ARTD10/PARP10 is the best-studied MARylating enzyme so far. ARTD10 shuttles from nuclei to the cytoplasm, thus targeting a wide array of proteins in the different compartments. ARTD10 has been involved in the modulation of mitochondrial function by means of silencing studies showing enhancement of mitochondrial oxidative capacity [130]. ARTD10 catalytic domain, a site for automodification, recruits GAPDH into intracellular compartments such as stress granules [131]. Two macrodomains in ARTD8 can selectively interact with ADP-ribosylated ARTD10 [132,133]. Human ARTD8 interacts with ARTD10 independent of automodification activity, while murine Artd8 macrodomains interaction with ARTD10 was found dependent on MARylation [134]. ARTD10 MARylates SRPK2, exportin-5 (XPO5), tubulin-β chain, pyruvate kinase (PKM), elongation factor 1-α1, UBEC3, and NF-κB essential modulator (NEMO, IKK-γ), a subunit of NF-κB transcription factor complex [133,135]: for these interactions, ARTD10 is considered to contribute to neurodegenerative disorders. Still, ARTD10 ADP-ribosylates PLK1, significantly inhibiting its kinase activity and oncogenic function in hepatocellular carcinoma (HCC) [121]. ARTD10 interacts with and MARylates aurora A, inhibiting its kinase activity [136,137]. Moreover, ARTD10 promotes cellular proliferation and alleviates replication stress [136]. Based on the chemical analysis of the reaction products, it was proposed that ARTD10 selectively modifies acidic amino acids. It was proposed that ARTD family members may also be capable of modifying serine residues proximal to lysine on account of the KS recognition motif [10,11]. ARTD10 contains several additional domains and motifs, including a RNA recognition motif (RRM), two functional ubiquitin interaction motifs (UIM), sequences capable of promoting nuclear targeting and nuclear export, and a small motif that mediates interaction with PCNA [138] and with ubiquitin receptor p62/SQSTM1 [133,139,140]. It is conceivable that some of these domains orient ARTD10 ADP-ribosylation toward specific substrates in different compartments. Furthermore, the ARTD10-mediated modification of proteins can regulate substrate function directly, as exemplified by glycogen synthase kinase 3ß (GSK3ß). GSK3ß is a well-investigated enzyme with established functions in WNT signaling, apoptosis, metabolism, neuronal development, immunity, and tumorigenesis [141,142,143,144,145,146,147,148]. ARTD10 ADP-ribosylates GSK3ß in vitro, reducing its kinase activity. This inhibition could not be overcome by increasing substrate concentration, implying that MARylation functions as an allosteric inhibitor of GSK3ß. ARTD10-modified GSK3β [87,149] has a regulatory role in type I IFN antiviral innate immune response, affecting the activation of IRF3 [150]. The nuclear transport protein RAN is also an ARTD10 substrate [87]. WRIP1 protein was used as a dual ARTD10/ARTD11 MARylation target in cell extracts [119,120].

Importantly, downregulation of ARTD10 induces glycolysis and mitochondrial fatty acid oxidation, which associates with a hypermetabolic cellular state [151]. Lastly, targeting ARTD10 may reduce the proliferation of cancer cells, as shown using antisense constructs or analyzing ARTD10 downregulated cell systems [137,152,153]. PARP10 deficiency produced severe developmental delay and DNA repair defect [152]. PLK1 inhibitors, alone or with NF-κB antagonists, were suggested as potential effective therapeutics for PARP10-expressing HCC [153]. Finally, ARTD10 can modify all four histones [140]. In Table 2 are described the ARTD10 interaction partners and MARylation effects.

#### 3.1.2. mART Inhibitors

Numerous research groups and drug discovery programs have been dedicated to inhibitors of ART enzymes [155]. Targeting ARTs has proven to be efficacious clinically, but the exploration of the therapeutic potential of ART inhibiting molecules has been largely limited by targeting the poly(ADP-ribose) generating PARP, including ARTD1 and ARTD2, as well as tankyrases [4]. Less attention has been put on the identification of selective inhibitors of mART. Nevertheless, considerable efforts have been made in order to deliver structure-based selective and potent drugs, which may be exploited for the treatment of pathological conditions, such as cancer, inflammatory diseases, as well as in buffering pathological NAD^+^ consumption.

For instance, the ARTD3 selective and cell-permeable ME0328, which displays >7-fold selectivity over ARTD1 and its nearest homologs [64], has been developed in Schuler laboratory, providing both a valuable tool to investigate ARTD3 functions in DNA damage repair and in delaying DNA repair in irradiated cancer cells. Similar approaches have led to the identification of the potent ARTD11/PARP11 inhibitor, which is greater than 200-fold selective over other mARTD family members [68]. By screening a collection of compounds for their ability to induce mitotic defects, AZ0108 was identified as a potent ARTD17/PARP6 inhibitor, which has been proved, leading to apoptosis in a subset of breast cancer cells in vitro and antitumor effects in vivo [69]. Several groups have contributed to identifying molecules with the potential to selectively inhibit ARTD10 [65,76]; of these, the most employed is the cell-permeable OUL35 [66]. However, additional compounds with submicromolar cellular potency have been additionally developed, such as a 3,4-dihydroisoquinolin-1(2H)-one that contains a methyl group at the C-5 position and a substituted pyridine at the C-6 position [67], and 4-benzyloxybenzimide derivatives [156].

Similarly, structure-based drug designing has led to the development of potent ARTD8/PARP14 inhibitors, such as the H10 showing >20-fold selectivity over ARTD1 [73] and a series of (Z)-4-(3-carbamoylphenylamino)-4-oxobut-2-enyl amides, the most potent of which was the compound 4t, that lacks selectivity against ARTD1, but displays >10-fold selectivity over ARTD5/PARP5a and >5-fold selectivity over closely related ARTD10 [74]. In addition, a series of diaryl ethers have been identified for their ability to discern between two closely related mARTDs, namely ARTD10 and ARTD8. Structure–activity studies identified compound 8b as a sub-micromolar inhibitor of ARTD10 with ~15-fold selectivity over ARTD8. By contrast, compounds 8k and 8m were discovered to have sub-micromolar potency against ARTD8 and demonstrated moderate selectivity over ARTD10. Importantly, all such compounds demonstrate selectivity over ARTD1 [77].

Finally, a first-in-class ARTD14/PARP7 inhibitor (RBN-2397) has been developed by Ribon Therapeutics, which already entered a phase 1 clinical trial (Identifier: NCT04053673) for patients with advanced or metastatic solid tumors. The rationale of this trial is based on ARTD14/PARP7 dependency of several cancer cells (such as lung cancer cells) for proliferation, especially of those cell lines with higher baseline expression of interferon (IFN)-stimulated genes. In particular, RBN-2397 appears to induce both cancer cell-autonomous and immune-stimulatory effects via enhanced IFN signaling [72].

In Table 1 are listed the known effects of mART inhibitors with a specific selectivity for members of the ARTD family, letting envisage their potential therapeutic application in human diseases.

### 3.2. Cholera-Toxin-Like Mono(ADP-Ribosyl) Transferases

ARTCs are able to MARylate protein substrates on arginine residues through N-glycoside bonds. Four ARTCs are expressed in humans (namely ARTC1, ARTC3, ARTC4, ARTC5), and six in mice (Artc1, Artc2.1, Artc2.2, Artc3, Artc4, and Artc5) [157]. The majority of mammalian ARTCs are glycosylphosphatidylinositol (GPI)-anchored proteins, with the exception of Artc5 that is a secreted enzyme [15,158]. ARTC1 is a GPI anchored protein facing the extracellular space, which modifies T-cell co-receptors and circulating hemopexin, a heme transport protein [158,159]. ARTC1 is also present on membranes of intracellular compartments and modifies Grp78/BiP in ER, which dissociates from stress sensors [160,161]. Among heat shock proteins, Hsp70-5 (HSPA5/BiP/GRP78) is localized at the endoplasmic reticulum facilitating transport and folding of nascent polypeptides into the ER lumen. Hsp70-8 (HSPA8, Hsc70, Hsp73, Hsc72) is the cognate Hsp70 family member that exhibits essential housekeeping functions, i.e., the folding of nascent polypeptides and misfolded proteins. Hsp70-9 (HSPA9, mortalin, GRP75, mtHsp70) is a mitochondrial Hsp70 isoform that bears a 46-amino acid target signal responsible for localization to the mitochondrial lumen. In CHO cells and human HEK293T and HeLa cells, BiP is ADP-ribosylated by hamster ARTC2.1 and by human ARTC1. BiP is ADP-ribosylated during ER stress, leading to a block in protein translation. In quiescent Swiss 3T3, Rat-1 cells, and mouse embryonic fibroblasts, BiP is ADP-ribosylated; however, when proliferation is induced, ADP-ribosylation is reduced. Mass spectrometry studies have led to identification of ADP-ribosylated residues, such as D78 and K81 of BiP, as well as D53 of Hsc70 in HeLa cells, and residue R50 of BiP and R346 of HSPA13 in murine skeletal muscle. R470 or R492 may be either ADP-ribosylated or may be AMPylated. ADP-ribosylated BiP is present in the lower-molecular weight fractions, indicating that ADP-ribosylation prevents BiP participation in the multi-chaperone complexes. Additionally, ADP-ribosylation is found only on the oligomeric form of BiP, which is the predominant form under the low protein-folding burden. Then, BiP is ADP-ribosylated during low protein production/low unprocessed protein states [162]. ARTC1 ADP-ribosylates integrin α7 (ITGA7) and regulates the binding of integrin α7β1 to laminin [159]. ARTC5 is found expressed as a secreted enzyme and probably MARylates itself on arginine; defensin HNP-1 inhibits hARTC5 auto-ADP-ribosylation and is not a substrate for ARTC5 activity [162].

### 3.3. Sirtuins with MARylating Activity

Sirt uses NAD^+^ to remove acyl groups (including acetyl, glutaryl, malonyl, succinyl, and lipoyl groups) from lysine residues to form 2′-O-acyl-ADP-ribose in protein deacylation, and, in the case of SIRT4, SIRT6 and SIRT7, in MARylation of protein targets, transferring ADP-ribose from NAD^+^ to Arg, Cys, Ser, or Thr residues of proteins [163]. SIRT4 ADP-ribosylates glutamate dehydrogenase (GDH) [164] in mitochondria. This activity opposes the effects of calorie restriction; thus, SIRT4 effectively antagonizes SIRT1. Furthermore, researchers ascribed to SIRT4 a negative effect on mitochondrial quality. The targeting of GDH inhibits the conversion of glutamate to α-ketoglutarate (α-KG), decreasing glutamine uptake, inhibiting cancer cell growth and interfering with epithelial to mesenchymal transition (EMT) in gastric cancer. Therefore, SIRT4 is considered a tumor suppressor [165,166]. SIRT4 was also associated with negative impacts on the mitochondrial quality and with aging [15]. It is possible that the antagonism with SIRT1 may increase cellular oxidative stress. α-KG upregulates H3/H4 histone acetylases and is required for α-KG-dependent N6-methyladenine demethylase ALKBH5, an RNA modifying enzyme; other Krebs cycle intermediates also show an inhibitory effect on epigenetic enzymes [167]. Importantly, the concentration of NAD^+^ in mitochondria needs to sustain SIRT4 activity; thus, NAMPT downregulation during aging may affect SIRT4 regulatory function.

**Figure 2:**
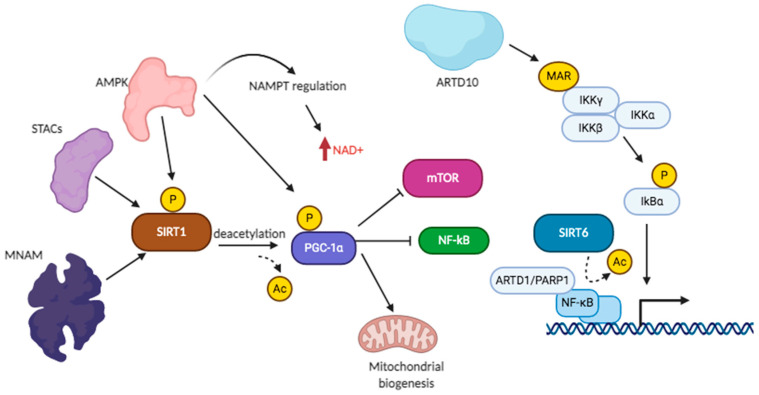
Sirtuin and ART signaling converging on NF-kB regulation.

SIRT6 and SIRT7 are localized in the nuclei. Overexpression of SIRT6 is found in skin cancer and in non-small cell lung cancer (NSCLC) with poor prognostic value, but in other types of cancers, it may be considered a tumor suppressor. SIRT6 is regulated by deacetylation nicotinamide phosphoribosyltransferase (NAMPT) activity and restores NAD(P)(H) pools in cancer cells [168]. SIRT6 has a role in the stabilization and phosphorylation of tau protein [169]. SIRT6 has been involved in genome integrity, DNA repair, energy metabolism and inflammation, and is found decreased during aging and cell senescence. SIRT6 was found to auto-ADP-ribosylate [170]. SIRT6 has ADP-ribosylation activity on ARTD1 on K521 [170], enhancing DNA repair, especially when phosphorylated by JNK on Ser10. SIRT6 ADP-ribosylates epigenetic enzymes [171] such as lysine demethylase JHDM1A/KDM2A, chromatin silencing factors such as nuclear co-repressor protein KAP1, regulating KAP1 interaction with HP1α and silencing of LINE1 retrotransposons [172,173]. SIRT6 ADP-ribosylates BAF170, activating the transcription of a subset of Nrf2 target genes, and this activity may sustain Nrf2-dependent boost of mitochondrial function [174]. Lamin A binds SIRT6 and promotes histone deacetylation [175], as well as SIRT6-mediated functions upon DNA damage, forming a multiprotein complex with RPA, Ku70, Ku80, proteins that bring together BRCA1, 53BP1, CDH4, and ARTD1. SIRT6 ADP-ribosylating activity induces activation of the p53- and p73-dependent apoptosis induction in cancer cells [176]. SIRT6, in particular the catalytically active form, associates in a phosphorylation-dependent mode with Ras-GTPase activator G3BP1 [177], with transcription factors NKRF, BCLAF1 and THRAP3, the telomerase regulator YLPM1, and the RNA polymerase complex factors XRN2 and COIL. SIRT6-deficient cell lines showed increased NF-kB activation and premature aging, linked to high H3 acetylation levels [178]. Signaling converging to NF-kB activation or inhibition [179], showing SIRT1 and SIRT6 regulatory roles interconnected with regulation by ARTD1 and ARTD10, has been schematically drawn, as shown in Figure 2.

Functional knockdown of SIRT6 results in tumor cell proliferation, invasive profile, and antiapoptotic effect [180]. When SIRT6 was overexpressed, reports observed suppression of NF-κB-mediated inflammatory responses, delaying cellular senescence [181]. SIRT6 promotes heterochromatin silencing at specific genomic loci, prevents genomic instability and telomeres dysfunction [175,180]. Reduced SIRT6 activity was linked to Hutchinson–Gilford progeria syndrome (HGPS), a human premature aging disorder, due to disrupted interaction with lamin A [182]. SIRT6 has been shown involved in liver disease, inflammation, and bone-related issue. Inhibition of SIRT6 by OSS-128167 blocked the expression of thermogenic genes and activation of white fat breakdown, showing that SIRT6/AMPK pathway increase energy consumption, insulin sensitivity and heat production, thereby alleviating metabolic disorders [183]. Treatment of osteosarcoma with SIRT6 inhibitors increased sensitivity to doxorubicin through increased damaged DNA [184]. Finally, the development of small molecules inhibiting specifically either deacetylation or MARylation may provide new clues on SIRT6 functions [185]. SIRT6 deacetylase activity has important effects in cells; therefore, it is difficult to assign the phenotype observed in SIRT6 silenced cells to a defect in MARylation or in deacetylation activity. Deacetylation of histone H3 on K9 and K56 by SIRT6 can block the transcription of GLUT1 and LDH by Hif-1α, inhibits the transcription activity of Myc on Lin28b, and that of NF-κB on survivin, while decreasing transcription of the pro-apoptotic *Bax* gene in HCC development; a similar regulation of histone H3 leads to block FoxO3 transcription and binding to SREBP1/2 and PCSK9 promoters, leading to metabolic regulation of lipogenesis [182]. Deacetylation of NF-kB and FoxO1 leads to their delocalization from nuclei to cytoplasm. Non-histone substrates and additional catalytic activities of SIRT6 have been reported, but these noncanonical roles remain enigmatic. Genetic studies showed critical SIRT6 homeostatic cellular functions and the need to find molecular pathways driving SIRT6-associated phenotypes. As for the physiological role, SIRT6 activity promotes increased longevity by regulating metabolism and DNA repair. In Table 3 are reported the known functions of SIRT6.

SIRT7 plays a key role in mitochondrial function and, in the liver, it regulates autophagy and the physiological response to calorie restriction [186]. SIRT7 has been involved in genome integrity and Non-homologous end-joining (NHEJ) DNA repair [187]. SIRT7 has auto-modification MARylation activity [181]. SIRT7 auto-modification occurs on several sites, as proteomic studies identified 7–8 MARylated peptides, modifying SIRT7 chromatin distribution. In the ELHGN catalytic motif, conserved among sirtuins, H187 recognizes acetylated substrates and is involved in deacetylation activity. H_187_ is oriented toward the NAD^+^-binding pocket and the main catalytic site, as the flanking residues E185 and N189. In SIRT6 and SIRT7, these flanking residues are faced in the opposite direction, toward the surface of the cavity, and both residues interact to form a loop. These residues are important for their role in the ADP-ribosylation reaction: E185 is the catalytic residue that initiates the reaction, whereas N189 acts as the first acceptor of the ADP-ribosyl moiety. SIRT7 shows nucleolar enrichment, and SIRT7 auto-modification attracts the ADP-ribose-binding macrodomain of histone H2A1.1 (mH2A1) and promotes the enrichment of mH2A1 in loci associated with metabolic genes [186].

A structure of bacterial Sirt bound to the acetylated +2 arginine peptide shows how this arginine could enter the active site and react with a deacetylation reaction intermediate to yield an ADP-ribosylated peptide [188]. These studies may allow differentiating the residues and structures performing deacetylation from those involved in MARylation.

### 3.4. Additional NAD^+^-Consuming Reactions

NAD^+^ is also consumed by NAD^+^ glycohydrolases [189], such as the NAD^+^ glycohydrolase/cluster of differentiation 38 (CD38), which catalyzes the hydrolysis of NAD^+^ and cyclic ADP-ribose (cADPR), thus affecting the pool of cellular NAD^+^ [190]. CD38 and sterile alpha and TIR motif-containing 1 (SARM1) are two ectoenzymes on plasma membranes with ADP-ribosyl cyclase/cyclic ADP ribose hydrolase activity [191]. CD38 is also inserted into intracellular membranes, facilitates autophagy, and has a role in autophagic fusion with lysosomes [192]. In the CD38 knockout mouse, NADase activity was absent in all compartments, from plasma membranes to nuclei [193]. CD38 was shown to process NAD^+^-capped RNA in vitro into ADP-ribose-modified-RNA and nicotinamide [194]. CD38 degrades NAD^+^ and also NMN and NADP, generating second messengers such as ADP ribose (ADPR), cADPR, and nicotinic acid adenine dinucleotide phosphate (NAADP). SARM1 is required for activation of injury-induced axon degeneration and facilitates mitophagy in depolarized mitochondria; thus, SARM1 may be involved in neuroprotection. NAD^+^ depletion can be rescued by increasing NMNAT activity. To protect from NAD^+^ consumption by SARM1 activity, cytosolic NMNAT1 was overexpressed, producing a beneficial effect that was dependent on NMNAT1 activity [195]. CD38 inhibitor 78c was administered to slow down the age-related NAD^+^ decline [196]: a therapy with 78c improved physiological parameters, such as glucose homeostasis, cardiac function, muscle architecture, and exercise capacity. The mechanisms of these antiaging effects are still to be identified. Table 4 shows the effects of NAD^+^ boosters in disease treatment and the therapeutic applications of various drugs influencing NAD^+^ levels and regulating NAD-dependent enzymes, and their beneficial effects. In Table 5 are presented several drugs involved in the regulation of NAD^+^ consuming enzymes such as CD38, in the activation of Sirt, and in the enhancement of NAD^+^ synthesis and NAM reutilization. NAM levels should be kept under a certain threshold in order to avoid an inhibitory effect of NAD^+^-dependent enzymes when NAMPT and other enzymes in the NAD^+^ salvage pathway are downregulated.

## 4. NAD^+^ Boosters and Therapeutic Role in the Treatment of Diseases

A large set of information can be found on the beneficial effects of sustaining NAD^+^ levels in disease states with NAD^+^ boosters [214,215,216] and supplementation of NAD^+^ precursors [198,217]. A clinical trial on healthy overweight adults to test the safety of NIAGEN (nicotinamide riboside chloride) was positively concluded [218]. NAD^+^ supplementation increases mitochondrial function, leading to a lifespan extension [198,199,217,218,219,220]. Boosting NAD^+^ through precursors such as NAM, NMN or nicotinamide riboside (NR) may increase longevity and prevent age-related diseases. For instance, Zhang and colleagues showed that NAD⁺ repletion enhances the life span of mice [220].

De novo NAD^+^ synthesis or increased availability of NAD^+^ precursors may thus support cognitive functions and lead to a decrease of Aβ amyloid fibrils [221,222,223,224,225]. Nicotinamide treatment preserved mitochondrial integrity in mouse models of AD [220] in cells with a functional NAD^+^ salvage pathway. NMN exogenously added to a mouse model of AD substantially decreased multiple AD-associated pathological characteristics [224,225]. NAD^+^ levels can affect inflammation, caloric restriction, exercise, DNA repair, longevity, and healthspan [207]. In a study on a coronavirus-dependent decrease in NAD^+^ levels, with altered expression of ARTD enzymes and NAMPT, authors recommended administration of NAD^+^ precursors to alleviate the inflammatory state of lungs [207,224]. Presently, it is known that NAD^+^ availability affects SIRT1, and this reflects on cellular metabolism through the SIRT1/AMPK/mTOR axis, but it can be speculated that most NAD^+^-dependent enzymes, including MARylating enzymes, may increase their activity and may better function when NAD^+^ levels are maintained elevated. ARTD family-specific mART inhibitors may pass the requirement for the potential application in therapy of human diseases and may be combined with approved NAD^+^ boosters, especially in conditions known to decrease the function of the NAD^+^ salvage pathway or with predominant glycolysis state or in the presence of mitochondrial dysfunctions. It can be expected that supplementation of NAD^+^ precursors may delay the onset and progression of ADP-ribosylation-linked disease states, as it has been done for metabolic and degenerative diseases with decreased NAD^+^ levels.

## 5. Conclusions

In this review, we shed light on the intimate connection between NAD^+^, NAD^+^-consuming enzymes, and mitochondrial well-functioning, highlighting the central role of these pathways in the development of chronic, metabolic and degenerative diseases. In the balance of produced and consumed NAD^+^ pools, the activity of NAD^+^ utilizing enzymes alters their bioavailability in intracellular and extracellular compartments, causing modifications to energy production and to metabolites that may lead to cell death. NAD^+^-dependent enzymes, including mARTs, may exert their activity only if NAD^+^ levels are maintained elevated. We reviewed the involvement of ARTDs, ARTCs, and MARylating Sirt in important cellular functions, from inflammation to immunity, from epigenetic modulation to chromatin accessibility, which may be affected when NAD^+^ levels are decreased. Various NAD^+^ precursors have shown beneficial effects in supplementing the NAD^+^ requirements, especially in disease states in which there is a major request by NAD^+^-dependent enzyme systems, while enzyme small molecule activators or regulators may become beneficial in various stresses and inflammatory diseases.

## Figures and Tables

**Table 1:** Selective inhibitors of ADP-ribosyl transferases (ARTs) and therapeutic potential.

Dug	Target Molecules	Therapeutic Applications	References
Olaparib, Rucaparib, Niraparib, Veliparib, Talazoparib	ARTD1/PARP1 and ARTD2/PARP2 inhibition and trapping on DNA	BRCA1, BRCA2, ATM, ATR, FANC, PALB2 mutated, CCDC6 inactivated, HR defective cancers; RNASEH2B deleted cancers; HER negative cancers, prostate cancer	[14,63]
PJ34	Broad inhibitors	Cardiomyocytes protection against oxidative stress, improvement of organ functionality; suppression of inflammatory responses, accelerated wound healing during burn injury; treatment of ALS	[58,59,60,61,62]
ME0328	ARTD3/PARP3	Exploits vulnerabilities in DNA repair in cancers	[64]
IWR1, AZ-6102	ARTD5 and ARTD6 (Tankyrase-1 and 2)	Wnt-driven cancers	[4]
3,4-dihydroisoquinolin-1(2H)-one and 4-benzyloxybenzimide derivates, OUL35	ARTD10/PARP10	Neurodegenerative disorders and cancer	[65,66,67]
ITK7	ARTD11/PARP11	Various cancers, induce enzyme dissociation from nuclear envelope	[68]
Ribon RBN-010860, AZ0108	ARTD17/PARP6	Colorectal and other cancers	[69,70,71]
Ribon RBN-2397	ARTD14/PARP7	Anti-proliferative, lung cancer	[72]
H10	ARTD8/PARP14	N.D.	[73]
4t and group of -4-(3-carbamoylphenylamino)-4-oxobut-2-enyl amides	ARTD8/PARP14	N.D.	[73,74,75]
8b diaryl ether 8k and 8m diaryl ethers	ARTD10/PARP10 ARTD8/PARP14	N.D.	[76,77]

**Table 2:** Known substrates and biological roles of ARTD10.

ARTD10 Substrate	Molecular Outcomes	References
Aurora Kinase A	Inhibition, suppression of metastasis	[136,137]
PCNA	Activation, DNA damage tolerance, replicative stress	[136,138]
Myc and Ran as nuclear substrates	Inhibition	[87]
GSK3β	Inhibition	[87,150]
Histone 3	Proliferation	[154]
Tubulin-β, Exportin-5, SRPK2, PKM, EF1-α1, UBEC3	N.D.	[133]
PLK1	MARylation inhibits activity and oncogenic function	[135]
NEMO (IKK gamma)	Inhibition of NF-kB, inhibition polyubiquitination, anti-inflammatory role	[132]
ARTD10	Alleviation of replicative stress and increase of EMT	[136]
ARTD10	Increases Warburg enzymes, glycolysis. Reduces oxidative stress, increases AMPK activity, reduces ATP levels, decreases cancer proliferation	[152,153]

**Table 3:** Substrates and biological roles of SIRT6.

SIRT6 Substrates, Partners, Co-Activators	Molecular Outcomes	Cellular Outcomes	References
SIRT6	N.D.	Induces p53-dependent apoptosis	[176]
PARP1	Recruitment of repair enzymes	Efficient DNA repair	[170]
KDMA2	Increases H3K36me2 at DNA damage sites to inhibit transcription and promote repair	N.D.	[172]
KAP1	Blocks KAP1 -HP1α binding, silencing of LINE1	N.D.	[173]
BAF170	Transcription of Nrf2 target genes	N.D.	[174]
Hif1α	Interaction and inhibition	Inhibition of mitochondrial respiration. Induces glycolysis, cell survival, cancer, ageing	[182]
Interaction with RELA /p65 NF-κB subunit	Attenuates NF-κB on promoters	Cell senescence, anti-apoptotic, insulin sensitivity	[178]
H3 de-acetylation	Inhibition GLUT1/LDH transcription by Hif-1α in White and brown fat cells	N.D.	[182]
N.D.	Inhibition of NF-κB transcription activity and suppression of Survivin gene expression in HCC	N.D.	[179]
Lamin A interaction	Induces Sir6 chromatin localisation and Histone deacetylation	N.D.	[175]

**Table 4:** NAD+ precursors, NAD+ salvage enzyme activators, and Sirt regulators and inhibitors with therapeutic potential.

Molecule	Activity	Cellular Outcomes and Therapeutic Application	References
Kynurenine, quinolinic acid	NAD^+^ precursor	Preserves mitochondrial integrity in mouse models of AD, Parkinson’s disease (PD), amyotrophic lateral sclerosis (ALS), Alzheimer’s disease (AD), Huntington’s disease (HD)	[190]
NMN	NAD^+^ precursors	Reverses JNK activity and progression of AD, age-related pathological processes, such as diabetes, ischemia-reperfusion injury, heart failure	[197]
NR, NRH	NAD^+^ precursors	Clinical trial of healthy overweight adults, AD protection through the induction of autophagy. They protect mice against a high fat diet, prevent age-related diseases and increase longevity	[26,45,198]
Nicotinic acid	NAD^+^ precursors	Treatment of tumor cells deficient in NAPRT	[55,62]
NAM	NAD^+^ precursors	Treatment of early AD	[199,200]
Salidroside	Activation of NAPRT and NAPRT signalling pathways	Protects PC12 cells from Aβ_140_ induced cytotoxicity; clears α-sinuclein aggregates by autophagy and mTOR regulation. Anti-apoptotic effect	[201]
1-methyl NAM	NAD^+^ precursors	Lifespan extension, mitohormetic signalling by H_2_O_2_. It protects from lipotoxicity in renal tubular kidney cells, protective compensatory response to injury in skeletal muscle	[49,50]
Sirtuin activating compounds, stilbenes, Flavonoids, fatty acids, chrysophanol	SIRT6 modulators	Alleviate Metabolic Syndromes	[183]
OSS-128167	SIRT6 inhibitor	Potentiates the anti-tumor effect of doxorubicin	[183,184]
Acetylated lysine-ADP-ribose conjugates	SIRT7 inhibitor	Histone H3K18 deacetylation and maintenance of oncogenic transformation	[202,203]

**Table 5:** Drugs regulating NAD+ synthesis and other enzymes in disease treatment and in combined therapies.

Molecule	Molecular Outcomes	Therapeutic Applications	References
Propargyl- Linked Bisubstrate analogues	NNMT inhibition/regulation	Increase of S-adenosyl methionine for epigenetic regulation; treatment of chronic obstructive pulmonary disease (COPD)	[204]
Substrate analogues	QPRT inhibition	Restore sensitivity to Imatinib in WT1 expressing K562 leukemic cells	[205]
Aminopropyl carbazole agents, P7C3-A20	NAMPT activation	Neuroprotection in Wallerian degeneration (WD) and SOD1 mouse model for ALS	[206]
FK-866, CHS-828	NAMPT inhibitor	Anti-tumor properties in various xenograft models. Stroke therapy, immunotherapy	[48,207,208]
FX-11	LDH inhibitor	Additive effect with FK-866 in reducing the growth of the human P493 B and MDA MB 231 cancer cell lines	[209]
NAD^+^ boosters or modulating enzymes involved in NAD^+^ generation or degradation; CD38i/ARTDi	Activation of myocyte survival enzymes, i.e., protein kinase B (Akt) and protein kinase C epsilon. They restore NAD^+^ myocardial concentration preventing decreases in myocardial contractility	Cardiovascular protection, atherosclerosis, coronary disease, acute myocardial infarction, heart failure	[210,211]
Rucaparib	PARPi	Antiviral response in mice	[88]
78c	CD38 inhibition, increases NAD^+^ levels	Improved physiological parameters, glucose homeostasis, cardiac function, muscle, exercise capacity, lipid homeostasis	[193]
Apigenin	Activation of Sirtuin and inhibition of CD38	Decrease global protein acetylation in obese mice	[212,213]

## Data Availability

The data presented in this study are available in the article.

## Abbreviations

PAR	poly(ADP-ribose)
MAR	mono(ADP-ribose)
ARTs	ADP-ribose transferases
NAD^+^	nicotinamide adenine dinucleotide
NA	nicotinic acid
NAM	nicotinamide
NMN	nicotinamide mononucleotide
NR	nicotinamide riboside
NRH	NR reduced form
NRK	nicotinamide riboside kinase
NRT	NR transporter
NNT	nicotinamide nucleotide transhydrogenase
NAMPT	nicotinamide phosphoribosyltransferase
NaNMN	nicotinic acid mononucleotide
NAPRT	nicotinic acid phosphoribosyltransferase
NMNATs	nicotinamide mononucleotide adenylyltransferase
ADK	adenosine kinase
CD38	NAD^+^ glycohydrolase (ADPR hydrolase)
CD73	ecto-5′-nucleotidase
NTPDase	nucleoside triphosphate diphosphohydrolase
ENPP	ectonucleotide pyrophosphatase/phosphodiesterase
MNAM	1-methylnicotinamide
Nnmt	nicotinamide-N-methyltransferase
QPRT	quinolinate phosphoribosyltransferase
QA	quinolinic acid
Kyn	kynurenine
HDAC	histone deacetylase
Sirt	sirtuin, NAD^+^-dependent HDAC
